# 

**Published:** 1890-04

**Authors:** 


					﻿Entered of the Austin, Texas, as Second Class MoR Matter
Vol. V
APRIL, 1890.
No. 19.
DANIEL'S
MEDICAL
JOURNAL
F. E. DANIEL., M. D., Editor and Publisher, AUSTIN, TEXAS.
Steam Book and Jor Printer, Austin, Texas
				

